# Post-traumatic extensive chronic osteomyelitis of skull vault: An illustrative case report

**DOI:** 10.12669/pjms.40.12(PINS).10977

**Published:** 2024-12

**Authors:** Maksalmina Reshtin, Ahmad Faeez, Haseeb Mehmood Qadri, Abdul Ghafoor, Ahtesham Khizar

**Affiliations:** 1 Maksalmina Reshtin, MBBS, Department of Neurosurgery Unit-I, Punjab Institute of Neurosciences, Lahore, Pakistan; 2 Ahmad Faeez, MBBS, Department of Neurosurgery Unit-I, Punjab Institute of Neurosciences, Lahore, Pakistan; 3 Haseeb Mehmood Qadri, MBBS, Department of Neurosurgery Unit-I, Punjab Institute of Neurosciences, Lahore, Pakistan; 4 Abdul Ghafoor, MBBS, MS (Neurosurgery), Department of Neurosurgery Unit-I, Punjab Institute of Neurosciences, Lahore, Pakistan; 5 Ahtesham Khizar, MBBS, FCPS (Neurosurgery), Department of Neurosurgery Unit-I, Punjab Institute of Neurosciences, Lahore, Pakistan

**Keywords:** Craniotomy, Cranioplasty, Osteomyelitis, Skull, Traumatic brain injuries

## Abstract

Chronic osteomyelitis of the skull base is a commonly reported pathology in existing scientific literature, but chronic osteomyelitis of the skull vault (COSV) is a rarely documented disease. We report the case of a 38 years old Afghan male with a presenting complaint of irregular swelling on the skull vault for six months. The patient had a history of head trauma one year back with a compound depressed fracture which had been surgically managed then. Physical examination revealed a 15 x 15 cm hard, immobile swelling with an old scar mark over the scalp. Neuroimaging was consistent with diffuse, bilateral frontoparietal swelling of bony origin, sparing underlying brain parenchyma. Surgically excision of the lesion was done through a bicoronal skin flap and cranioplasty done at the same time. Histological findings of the specimen reported chronic osteomyelitis. However, microbiology revealed no growth in culture. Patient was discharged on the second postoperative day uneventfully. This case turns minds into keeping skull vault osteomyelitis as differential diagnosis besides other spontaneous bony lesions; e.g. fibrous dysplasia, osteoma and giant cell tumours.

**Abbreviations:**
COSV: Chronic osteomyelitis of skull vault,
CT: Computed Tomography,
MRI: Magnetic Resonance Imaging,
PMMA: Polymethylmethacrylate,
SBO: Skull Base Osteomyelitis.

## INTRODUCTION

Cranial osteomyelitis is an uncommon life-threatening condition which can involve different parts of skull; mainly the base of skull (anterior, middle and posterior) and rarely cranial vault.^1^ Cranial osteomyelitis has a predilection for male gender (79.9%), immunocompromised, overweight and old age population. Diabetes mellitus is the most common comorbid condition.^1^, The most common causes of osteomyelitis in developing countries are paranasal sinusitis, traumatic head injury and scalp infections, while in developed countries post-surgical infections remain the main cause.^1^

### Staphylococcus aureus:

It is the most common organism in post-traumatic and anterior skull base osteomyelitis (SBO), while *Pseudomonas aeruginosa* is more frequently reported in middle and posterior SBO.^1^, Patients present with various nonspecific clinical conditions, with headache being the most common symptom seen in 73% of cases. Other complications include sixth and seventh cranial nerve palsy (78%), meningitis (63%), cerebral venous thrombosis (44%) and cerebral infarction (34%).^2^ Management includes prompt causative agent detection, broad spectrum antibiotics for eight to twenty weeks and aggressive surgical debridement. Prognosis is better with early diagnosis, surgical excision and complete antibiotic therapy.^1^,^2^

SBO is the condition that is encountered commonly, especially in low-middle-income countries, but skull vault osteomyelitis is a rare condition. To the best of our elaborate literature search using PubMed and Google Scholar, this is the first case of post-traumatic chronic osteomyelitis of skull vault (COSV) with a benign swelling on presentation.

## CASE PRESENTATION

**Fig.1:**
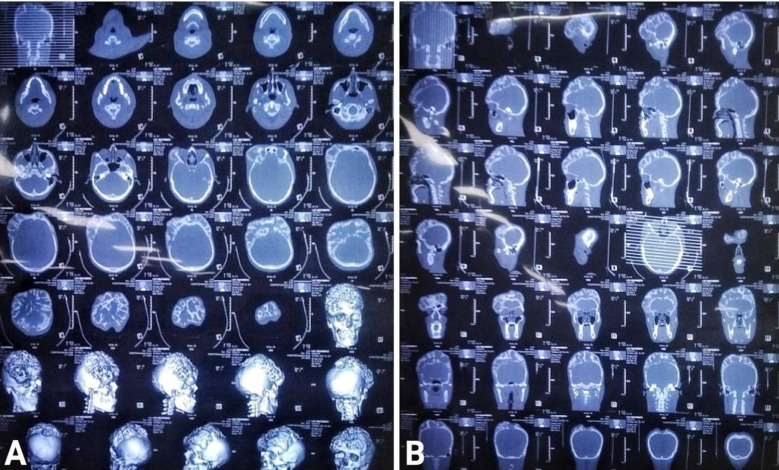
Computed Tomography (CT) scan of the lesion, A: Bone window axial view with 3D skull reconstructions showing texture changes and abnormal growth of bilateral frontoparietal bones, B: Bone window sagittal and coronal views showing extensive growth of bilateral frontoparietal bones.

**Fig.2:**
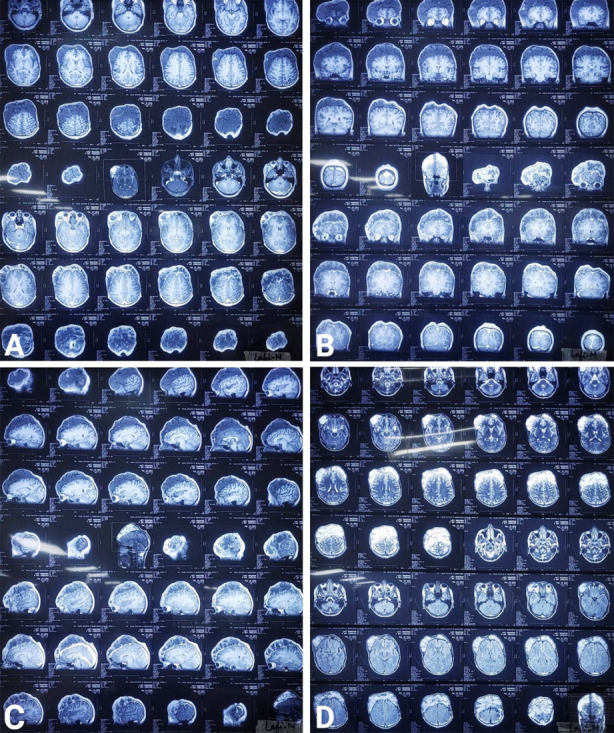
Magnetic Resonance Imaging (MRI) of the lesion, A: T1WI & T1C+ axial views showing bilateral frontoparietal hypointense lesion on T1WI and heterogeneously enhancing lesion on T1C+, B: T1WI & T1C+ coronal views showing the same findings, C: T1WI &T1C+ sagittal views showing the similar findings, D: T2WI & FLAIR axial views showing hyperintense lesion on both views.

**Fig.3:**
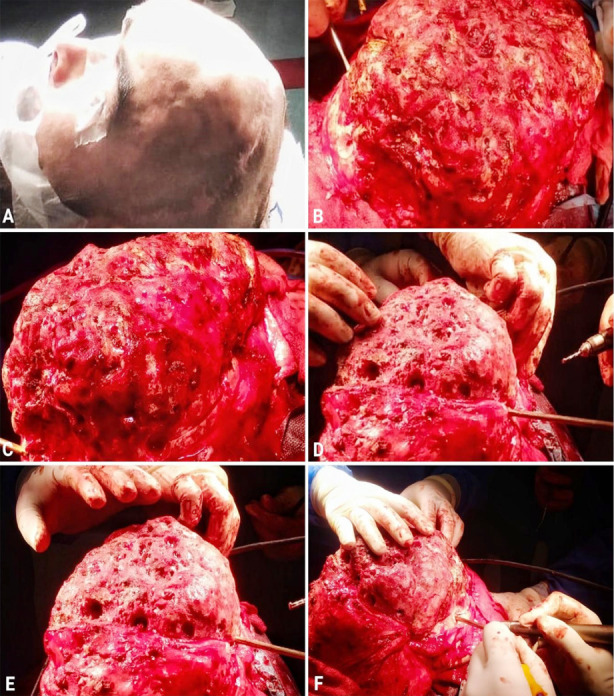
A: Preoperative view of the head, B&C: Intraoperative views of the lesion, D&E: Multiple burr holes made, F: Bone cutting with high-speed drill system. Fig.5: Histological examination sections reveal: A) Viable and necrotic bony fragments with marrow elements. Mild chronic inflammation is evident, B) Fibrocollagenous tissue and bony tissue fragments. Sheets of macrophages are also seen. Focal area of calcification is noted.

**Fig.4:**
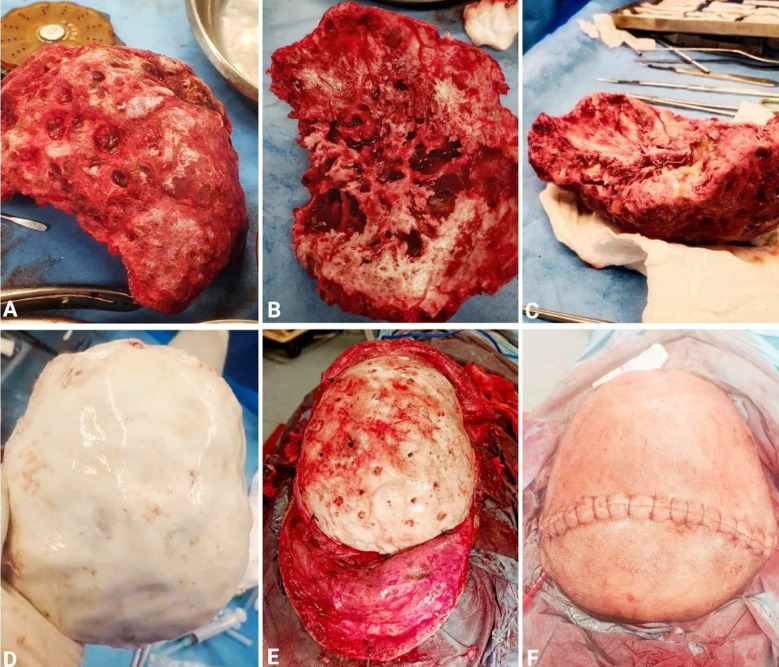
A: Superior, B: Inferior & C: Lateral views of the removed skull showing multiple cavities inside the bone, D: Custom made PMMA cranioplasty implant, E: Cranioplasty implant in place, F: Postoperative view at closure.

A 38-year-old male Afghan native was sent to our outpatient clinic after complaining of frontal irregular swelling for six months. There were no further related complaints. On examination, there was a bony, hard, irregular-shaped swelling across bilateral supraorbital ridges anteriorly, extending to the mid scalp posteriorly, measuring approximately 15 × 15 cm in size and 3 - 4 cm in height (Fig.3A). There were no apparent pulsations or discharging sinuses. Swelling was firm and somewhat tender, with a normal temperature, non-reducible, non-compressible, and non-mobile in any direction. The cough impulse was negative. The patient had a history of head trauma one year ago, with a compound skull fracture and underlying hematoma, for which he underwent a craniotomy and evacuation in his home country. Computed tomography (CT) and Magnetic resonance imaging (MRI) are shown in Fig.1 (A&B) and 2 (A, B, C&D). CT shows texture changes and abnormal growth of bilateral frontoparietal bones whereas MRI with contrast shows extensive heterogeneously enhancing lesion over bilateral frontoparietal region. Based on the history, physical examination, and imaging findings, post-traumatic fibrous dysplasia and bone tumour were the differentials under consideration. Surgery was intended for cosmetic and diagnostic purposes. During surgery, a bicoronal skin flap was raised and a bony lesion was excised, followed by cranioplasty using bone cement (Fig.4 D&E), also known as polymethyl methacrylate (PMMA). A 15 × 15 cm hard bone lesion with several tiny cavities filled with soft greyish jelly-like substance was discovered intraoperatively as shown in Fig.3 (B, C, D, E&F) and 4 (A, B&C). It was an incredibly fragile bone with adhesions to the dura in some spots and widespread bleeding from the dura. A complete excision of the bony lesion was performed, as well as cranioplasty for the bony defect, and two subgaleal drains were placed (Fig.4 F). The drains were withdrawn after one day, and the patient was discharged on the second postoperative day in a stable condition. He was prescribed intravenous antibiotics and asked for follow-up with an acquaintance of Neurosurgery in his own country Afghanistan.

### Findings of histological examination:

### Gross:

Specimen was preserved in formalin in two containers: A): Bone: The specimen consisted of multiple bony pieces of tissue measuring 11.5x8.5x1.5 cm in aggregate. B): Soft Tissue: The specimen consisted of multiple soft and bony pieces of tissue measuring 5.5x4.5x1.0 cm in aggregate.

### Microscopy:

**Fig.5:**
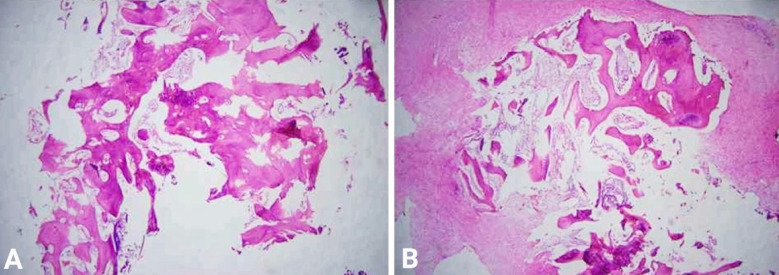
Histological examination sections reveal: A) Viable and necrotic bony fragments with marrow elements. Mild chronic inflammation is evident, B) Fibrocollagenous tissue and bony tissue fragments. Sheets of macrophages are also seen. Focal area of calcification is noted.

A): Histological examination of sections revealed viable and necrotic bony fragments reveal marrow elements. Mild chronic inflammation is evident. (Fig.5A) No granuloma or malignancy was seen. B): Histological examination of sections revealed fibrocollagenous tissue and bony tissue fragments. Sheets of macrophages were also seen. Focal area of calcification was noted. (Fig.5 B) No granuloma or malignancy was seen.

### Diagnosis:

A): Bone: Chronic osteomyelitis - No features of osteoid osteoma or chondrosarcoma were seen. B): Soft Tissue: Fibrocollagenous tissue and bony tissue fragments with focal calcification.

### Findings of microbiology:

No growth was seen in culture after 48 hours.

### Consent for Publication:

Consent was obtained from the patient for the publication of this case report and the accompanying images.

## DISCUSSION

The gross osteonecrosis of the skull bone along with invasion into the bone marrow cavity and extra-osseous environment constitutes to form the the picture of COSV.^3^Mortazavi et al. classify the causes of cranial osteomyelitis on the basis of its origin - sinorhino-otogenic and non-sinorhino-otogenic causes. It can affect children and adults both, post-traumatic cranial osteomyelitis, a non-sinorhino-otogenic causes, is more common among children than adults.^1^

A recently reported study by Das et al. from India documents that male gender was more commonly affected by SBO and the mean age of included patients at presentation was 56.9 ± 10.7 years.^2^This gender predilection is consistent with the case under study. However, the young age at presentation in our patient is attributed to a road traffic accident which could occur irrespective of the age.

A case report documented in Italy on COSV in 2019 also states the 45-year-old female had the complaints of headache and scalp wound after a road traffic accident. However, our patient did not have a history of a fever, headache or scalp wound which are the usual manifestations at presentation.^4^.^5^ This implies that it is possible for chronic osteomyelitis to present without systemic signs of inflammation and as a local pathology.

An Indian study by Das et al. both implicate diabetes mellitus as the most common comorbid condition in patients with SBO, that is 46.62% and 90%, respectively in their patients.,^2^ There were no identifiable medical comorbidities in our patient, except the history of surgery itself a year back.

CT scan is not diagnostic of active osteomyelitis in post-traumatic and post-surgical cases. The existing English scientific literature advocates MRI over CT. MRI plays a key role in defining bone and soft tissue changes of skull vault.^1^ COSV may present as diffuse osteolysis or diffuse bone thickening.^6^ Similarly, bone thickening with hypointensity on T1WI and hyperintensity on T2WI were notable features in our patient on MRI. Hyperintensity on T2WI was suggestive of chronic osteomyelitis in other cases too.^4^.^5^

Incomplete penetrance of antibiotics into the medullary cavity and wide range of bacterial resistance makes the treatment of chronic osteomyelitis difficult to manage medicinally.^3^ Yang et al. propose that the ideal antibiotics to treat post-traumatic osteomyelitis must possess the qualities of wide antibacterial target, maximum safety, least allergic properties and good water-solubility, but not all qualities are possessed by a single antibiotic. Gentamicin and Vancomycin are the most widely used ones. We opted for wide surgical excision for the same reasons, as well as to improve the cosmetic deformity in our young patient. Augmentation with PMMA is being practiced for the last 40 years and so we did cranioplasty using PMMA.^3^

Culture negative SBO was seen in 26.64% and 39% patients in the recently published studies.^2^ Case reports on COSV often have inconclusive cultures,^4^.^5^ but cases of Actinomyces and Klebsiella have also been reported.^6^.^7^

## CONCLUSIONS

COSV is one of the rare infections involving the head and neck. It can affect patients of all ages. Scalp swellings without the typical manifestations of headache, fever and scalp ulcers may also present as COSV. Surgical excision with concomitant cranioplasty in cases of gross involvement of cranial vault and chronicity shall be considered.

### Authors’ Contribution:

**MR and AF:** Data acquisition, Manuscript writing and Literature review.

**HMQ:** Literature review, Data collection, Manuscript writing and editing and, Critical review.

**AG:** Data acquisition, Supervision and Review.

**AK:** Conception and Design of study, Manuscript writing and Manuscript editing.

All the authors have read and approved the final manuscript and are responsible and accountable for the accuracy and integrity of the work.
